# Proxy immunization enabling yellow fever vaccination after thymectomy

**DOI:** 10.1038/s41541-026-01520-x

**Published:** 2026-06-29

**Authors:** Emilie C. Rijnink, Muriel Aguilar-Bretones, Rory D. de Vries, Sharon Veenbergen, Daniël G. Aynekulu Mersha, Corine H. Geurts van Kessel, Marion P. G. Koopmans, Pieter A. van Doorn, Lennert Slobbe, Rik A. Brooimans, Gijsbert P. van Nierop, Casper Rokx

**Affiliations:** 1 Department of Internal Medicine, Section Infectious Diseases, Erasmus University Medical Center, Rotterdam, Netherlands; 2 Department of Medical Microbiology and Infectious Diseases, Erasmus University Medical Center, Rotterdam, Netherlands; 3 Department of Viroscience, Erasmus University Medical Center, Rotterdam, Netherlands; 4 Laboratory Medical Immunology, Department of Immunology, Erasmus University Medical Center, Rotterdam, Netherlands; 5 Department of Neurology, Erasmus University Medical Center, Rotterdam, Netherlands

**Keywords:** Diseases, Immunology, Medical research

## Abstract

Yellow fever (YF) vaccination is generally contraindicated in individuals with thymic disorders because of the risk of YF vaccine–associated viscerotropic disease (YEL‑AVD). As YF transmission expands and global travel increases, protection may still be indicated for selected patients with prior thymectomy. We report a 25‑year‑old woman with childhood thymectomy for acetylcholine receptor antibody–positive generalized myasthenia gravis, now in long‑term remission without immunosuppression, who required YF prevention. Immune competence was evaluated using monitored orthoflaviviral proxy immunization with the live‑attenuated tetravalent dengue vaccine (TAK‑003). She experienced mild reactogenicity and developed dengue‑specific antibodies and T‑cell responses, with preserved B-cell maturation. After confirming orthoflaviviral immune competence, she received YF‑17D‑204 vaccination, which was well tolerated and induced transient viremia, seroconversion, and functional YF‑specific B‑ and T‑cell responses. This case demonstrates the feasibility of an individualized, immune‑guided approach to safely administering YF vaccination after thymectomy and highlights the need for broader clinical confirmation.

## Introduction

The yellow fever (YF) vaccine, a live-attenuated virus vaccine used since 1937, is generally safe and well-tolerated^1^. In 2001, however, YF vaccine-associated viscerotropic disease (YEL-AVD) was recognized as a rare but often fatal complication resembling wild-type YF^2,3^, occurring in around 0·8 per 100,000 vaccinees^4^. Because of the limited number of cases, identifying risk factors has been difficult^5^. In a 2004 overview, four (17%) of 23 vaccinees with YEL-AVD had undergone thymectomy for thymus disease, suggesting a particular vulnerability^2,3,6^. Since then, only two additional YEL-AVD cases were reported with confirmed thymus pathology^7,8^. These reports nonetheless led to an absolute contraindication in guidelines for YF vaccination in all persons with thymus disorders or prior thymectomy based on a presumed loss of thymus (T)-cell mediated control of vaccine viremia^9,10^.

Recent YF outbreaks in the Americas^11^ and vector expansion driven by climate change mean that more immunocompromised patients, currently ineligible for YF vaccination, may face exposure to this potentially lethal viral infection. Moreover, unvaccinated travelers face occupational or geographic restrictions where proof of YF vaccination is mandatory. Given the rarity of YEL-AVD, increasing YF endemicity, and a growing population with immunodeficiencies, defining subgroups in whom YF vaccination can be safe is increasingly warranted.

To address this clinical need in thymectomized individuals with possibly altered T-cell repertoire formation, a novel strategy could be a monitored proxy-vaccination strategy using the live-attenuated dengue virus (DENV) vaccine TAK-003, which received prequalification by the World Health Organization (WHO) as of May 2024. This closely related orthoflavivirus vaccine could make a suitable proxy, as no DENV vaccine-induced viscerotropic disease has been reported. YF vaccination is safe in DENV pre-immune individuals, and it provides a relevant added protection to co-circulating orthoflaviviruses in overlapping geographic regions^5,12,13^. Here, we describe a case of a DENV and YF virus naïve and otherwise healthy case with prior thymectomy, where this approach enabled evaluation of adaptive immune competence before safely administering YF vaccination, providing insights into a clinical model that may support individualized decision making on YF vaccine use rather than categorical exclusion after thymectomy.

## Results

### Case presentation

A 25-year-old female was referred to the infectious disease outpatient clinic by her neurologist to assess whether YF vaccination was required for her career in aviation. She was diagnosed in 2011 with acetylcholine receptor antibody-positive myasthenia gravis at age 11 based on an 8-day history of sudden-onset left-sided ptosis, which exacerbated during the day. Physical examination then confirmed a left-sided ptosis to the apical pupil with provoked diplopia on prolonged fixed lateral eye movement. The symptoms abated by neostigmine administration. After the initiated pyridostigmine treatment, symptoms evolved to diplopia with speaking and swallowing difficulties, and progressive upper and lower extremity weakness that interfered with daily activities and sports. Neither the progressively increased frequency and dosage of pyridostigmine nor the initiation of prednisone at 60 mg once daily in 2012 resulted in clinical improvement. Given ineffective medical treatments, she underwent thymectomy at age 13. The patient underwent robot-assisted thymectomy in 2012 via a right pleurectomy approach. The surgical report notes a thymus of normal size with a generally expected shape except for 2 more proximally extended horns. Gross pathological examination revealed an 8 × 6 × 0.7 cm fibrous-adipose specimen containing white, lobulated glandular parenchyma without nodules. Histologic analysis demonstrated preservation of the lobulated architecture, with lymphoid tissue embedded in mature fatty tissue. The lymphoid tissue and epithelial dendritic networks were intact, and occasional Hassall’s bodies were observed. No atypical lymphoid epithelial infiltration was noted, and the pathologist did not find evidence of a thymoma.

The thymectomy subsequently led to clinical remission of symptoms that was sustained after interruption of pyridostigmine and prednisone tapering in 2014, although serum anti-acetylcholine receptor antibodies were detected both before thymectomy in 2011 (12·7 nmol/L) and after in 2013 (11·9 nmol/L) and 2014 (18·4 nmol/L). Anti-muscle-specific kinase antibodies were not detected. Due to mild residual diplopia that was provokable during infections, pyridostigmine was restarted in 2016. Clinical remission was stable for the subsequent 8 years until referral in 2024 to the infectious disease unit for the YF vaccination request. At that time, she had only very minimal provokable weakness of facial muscles and diplopia in extreme fixed lateral eye positions on pyridostigmine 100 mg QID, and she was without immunosuppression. Her childhood vaccinations were uneventful, and she had no severe or recurrent infections. The indication for YF vaccination was carefully weighed against the risk of YEL-AVD. Given the patient’s preference and long-term stable myasthenic control without immunosuppression, the multidisciplinary team chose a clinical strategy to use a live-attenuated DENV vaccine as a proxy vaccination to assess safety and quality of adaptive immune responses to a related orthoflavivirus first to guide the decision on YF vaccination. This study adheres to the Declaration of Helsinki. Institutional review board approval was not required because this single-case report describes individualized clinical treatment under the Dutch Medical Treatment Contracts Act (WGBO). Written informed consent was obtained, and all data were anonymized.

### Baseline immune function

**Fig. 1:**
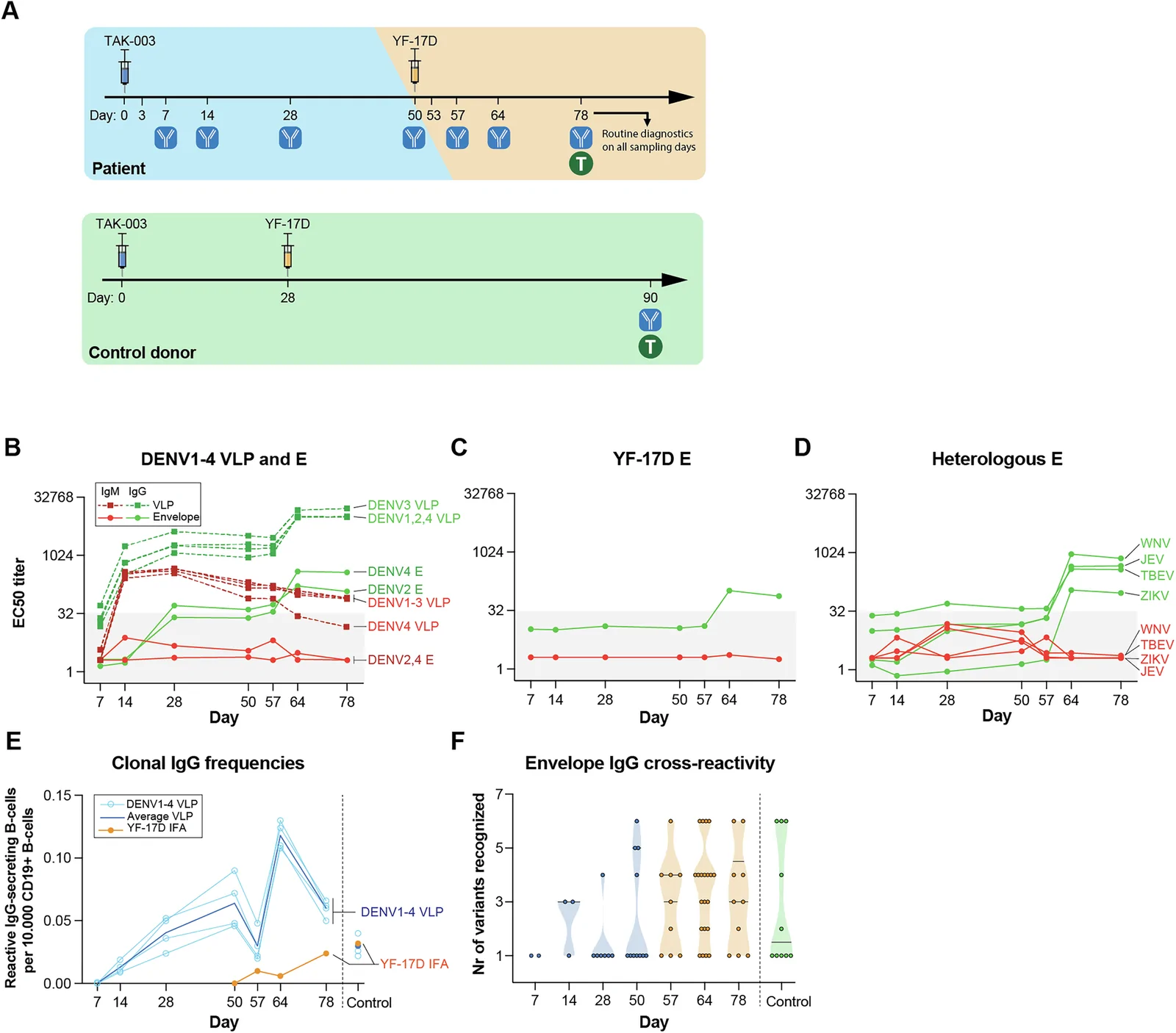
Orthoflavivirus-specific IgG titers and B-cell frequencies. **A** Schematic overview of vaccination and sampling time points for the thymectomy patient and control. Serology, B-cell analyses (Y), and T-cell analyses (T) were performed on the indicated time points. **B** The 50% effective concentration (EC50) serum titers for IgM (red) and IgG (green) binding dengue virus serotype 1-4 (DENV1-4) virus like particles (VLP; squares and dotted lines) and DENV2/4 envelope (E; circles and continuous lines). A positivity cut-off of EC50 = 32 is indicated with a grey shade. **C** Serum EC50 titers of IgM and IgG binding YF virus and **D** West-Nile (WNV), Zika virus (ZIKV), tick-borne (TBEV) and Japanese encephalitis virus (JEV). **E** The frequency of DENV1-4 VLP and YF-17D-specific clonal IgG and **F** the number of different orthoflavivirus envelopes that each clone recognizes per time point is shown for the thymectomy patient and control.

The timeline is provided in Fig. 1A showing Day 0 (D0) that defines baseline before vaccinations, where normal serum immunoglobulins, CD4+ and CD8 + T-cell and B-cell counts, including naive and memory subsets, were detected (Table 1). Serum IgG antibodies were detected against varicella zoster virus, measles virus, and rubella virus, but were absent against herpes simplex virus and cytomegalovirus. Antigen-independent and -dependent stimuli elicited normal T-cell activation as expected for a 25-year-old woman (Supplementary Table 1). No auto-antibodies were detected by Luminex to interferon-γ, interferon-α, and the interleukins 6, 17 A, 17 F, and 22 (mean fluorescence intensity below 1000 limit of detection, above 5000 indicating a positive result), unlike some previously described YEL-AVD cases^14^.

**Table 1:** General immune phenotyping at baseline and after a single dengue virus (DENV) and yellow fever virus (YF) vaccination

	DENV vaccination (TAK-003)		YF vaccination (17D-204)			
Days post DENV/(YF) vaccination	D0 (administration)	D14	D50 (0) (administration)	D57 (7)	D64 (14)	
						Reference
Leukocytes (*10^9/L)	6.1	4.0	5.6	5.7	7.0	3.5-10.0
-Lymphocytes	35.9%	48.1%	27.6%	32.3%	19.4%	
-Monocytes	1.2%	7.4%	n.a.	n.a.	1.2%	
-Granulocytes	62.9%	44.5%	n.a.	n.a.	79.4%	
IgG (g/L)	14.3					7.0-16.0
IgA (g/L)	3.12					0.76-3.9
IgM (g/L)	0.7					0.45-2.3
T-cells (*10^9/L)	1.71	1.29	1.34	1.47	1.17	0.7-2.1
CD4 + T-cells (*10^9/L)	1.26	0.95	0.95	1.08	0.81	0.3-1.4
CD8 + T-cells (*10^9/L)	0.35	0.28	0.33	0.32	0.30	0.2-0.9
CD4:CD8 ratio	3.5	3.4	2.9	3.3	2.7	
B-cells (*10^9/L)	0.22	0.13	0.14	0.22	0.19	0.1-0.5
NK-cells (*10^9/L)	0.46	0.23	0.32	0.17	0.13	0.09-0.6
Transitional B-cells (cells/µL)	9	7	11	20	17	3-50
Naive mature B-cells (cells/µL)	107	56	67	91	96	57-447
Natural effector B-cells (cells/µL)	35	16	24	41	31	9-88
Memory B-cells (cells/µl)	42	21	22	40	33	13-122
Naive CD4 + T-cells (cells/µl)	666	495	486	533	348	74-1173
Central memory CD4 + T-cells (cells/µl)	557	411	446	513	448	117-886
Effector memory CD4 + T-cells (cells/µl)	48	34	36	42	37	14-500
Naive CD8 + T-cells (cells/µl)	193	177	205	198	166	29-737
Central memory CD8 + T-cells (cells/µl)	106	96	78	98	108	54-453
Effector memory CD8 + T-cells (cells/µl)	38	21	25	22	18	2-515

*D* day of study, *DENV* dengue virus, *IgG* immunoglobulin G, *IgA* immunoglobulin A, *IgM* immunoglobulin M; *n.a.* not assessed, *NK* natural killer, *YF* yellow fever virus.

### Effective proxy orthoflaviviral vaccination

To safely assess immune responsiveness to a related orthoflavivirus vaccine while bypassing the contraindicated YF viral replication machinery, the live-attenuated tetravalent DENV vaccine (TAK-003, Qdenga®) was administered. TAK-003 was chosen over alternative orthoflavivirus options because it utilizes an attenuated dengue virus serotype 2 (DENV-2) backbone rather than a YF genetic scaffold, eliminating the risk of viscerotropic disease. Furthermore, unlike the first-generation dengue vaccine (CYD-TDV, Dengvaxia®), TAK-003 features a long-term clinical safety profile that does not place dengue-naive individuals at an increased risk of antibody-dependent enhancement (ADE)^15,16^. In healthy vaccinees, this platform induces a self-limiting low-level viremia with IgG seroconversion within 4 weeks^16,17^. Our DENV-naive patient experienced mild, self-limiting reactogenicity (local pain, myalgia, fatigue) without fever or serious adverse events following a single TAK-003 dose. Transient, low-level viremia was observed, followed by the induction of dengue virus–specific immunoglobulins (Table 2). D28 whole blood flow-cytometry showed that 1·97% of the total CD8 + T-cell population was dengue virus-specific with upregulated activation-induced markers (CD69 and CD137) after stimulation with an HLA-class I-restricted DENV peptide pool (Mabtech®). Given the mild reactogenicity and normal functional adaptive humoral and T-cell responses with rapid orthoflaviviral control, we proceeded to YF vaccination in shared decision with the patient.

**Table 2:** Viral kinetics and immunogenicity after dengue virus (DENV) and yellow fever virus (YF) vaccination

DENV vaccination					
Days post DENV vaccination	D0	D3	D7	D14	D28
DENV RNA PCR (CT)^a^	neg	neg	30.8 (+)	31.5 (+)	neg
DENV NS1 antigen^b^	neg	neg	neg	neg	neg
DENV IgM^c^	neg	neg	neg	9.56 (++)	5.63 (+)
DENV IgG^c^	neg	neg	neg	1.17 (+)	1.34 (+)
**YF vaccination**					
**Days post YF/(DENV) vaccination**	**D0 (50)**	**D3 (53)**	**D7 (57)**	**D14 (64)**	**D28 (78)**
YF RNA PCR (copies/mL)^d^	neg	neg	840 (+)	neg	neg
YF IgM (titer)^e^	1:10 (+)	1:10 (+)	1:10 (+)	1:100 (++)	1:10 (+)
YF IgG(titer)^e^	neg	neg	neg	≥1:1000 (++)	≥1:1000 (++)
YF PRNT (titer)^f^	neg	neg	neg	≥1:387 (++)	≥1:512 (++)

^a^For the DENV serotypes 1–4 PCR, cycle‑threshold values < 45 are interpreted as positive.

^b^For the DENV NS1 ELISA, ratios < 0.5 are interpreted as negative, ratios ≥ 0.5 to 1.0 as borderline, and ratios ≥ 1.0 as positive.

^c^For the DENV‑specific IgM and IgG ELISA, ratios < 0.8 are interpreted as negative, ratios ≥ 0.8 to 1.1 as borderline, and ratios ≥ 1.1 as positive.

^d^For the quantitative YFV PCR assay, the lower limit of detection is 750 copies per milliliter.

^e^For the YFV immunofluorescence assay, titers > 1:10 are interpreted as positive.

^f^For the YFV PRNT, titers ≥ 1:8 are interpreted as positive.

*CT* denotes cycle threshold, *D* day of study, *DENV* dengue virus, *ELISA* enzyme‑linked immunosorbent assay, *IgG* immunoglobulin G, *IgM* immunoglobulin M, *neg* negative result, *NS1* nonstructural protein 1, *PCR* polymerase chain reaction, *PRNT* plaque‑reduction neutralization test, *YF* yellow fever, *YFV* yellow fever virus.

### Safe YF vaccination-induced immunogenicity

On D50 after DENV vaccination, the YF-17D-204 (Stamaril®) vaccine was administered. The used YF-17D-204 batch average viral titer of 39,824 units/dose exceeded the manufacturer-specified required ≥1000 units/dose. No reactogenicity was observed, and transient low-level viremia with seroconversion occurred (Table 2). This pattern was comparable to healthy YF-17D-204 vaccinees, in whom transient viremia followed by detectable serum YF virus-specific IgG occurs within 2 weeks^18,19^.

Given orthoflaviviral antigenic relatedness and antibody cross-reactivity, we assessed serum antibody kinetics and breadth via IgM and IgG half-maximal effective concentration (EC50) titers against envelope and virus-like particles of DENV serotypes 1-4, YF, and heterologous orthoflavivirus lineages. After D0, DENV vaccination induced DENV antibodies (Fig. 1B), and after D50 17D-204 vaccination boosted IgG binding to YF viral envelope (Fig. 1C), heterologous orthoflaviviruses (Fig. 1D), and DENV-like particles, which plateaued from D64 onwards. SARS-CoV-2 spike and tetanus toxoid controls showed stable specific IgG titers at all timepoints (data not shown). Furthermore, DENV1-4 NS1 IgM/IgG serology data (Supplementary Fig. 1) demonstrated a distinct DENV-2 NS1 IgM and IgG response, as compared to other DENV and heterologous orthoflavivirus NS1.

### Broad clonal YF virus-specific B-cells responses

Because sequential flavivirus exposures can drive cross-reactive B-cell activation that reflects both immune competence and potential interference, we monitored the establishment and evolution of orthoflavivirus-specific IgG responses. To exclude whether thymectomy impaired such cross-reactivity, we generated clonal CD19 + B-cell profiles to quantify and distinguish YF virus-specific, heterologous orthoflavivirus-specific, and cross-reactive memory B-cell-derived IgG after both vaccinations, using control samples from a 38-year-old male with similar heterologous vaccinations (Fig. 1A).

TAK-003 vaccination induced DENV-specific B-cell-secreted IgG clones reaching 0·064% out of total CD19 + B-cells at D50. Of note, no YF virus-binding IgG-secreting CD19 + B-cell clones were detected then (Fig. 1E). Following D50 YF-17D-204 vaccination, the frequency of DENV-specific IgG-secreting CD19 + B-cell clones showed a temporary decline, followed by a steep boost alongside the emergence of YF-specific IgG-secreting CD19 + B-cell clones. At D78, 0·024% of IgG CD19 + B-cell clones reacted to YF-17D, comparable to control levels (0·03% YF-17D-specific IgG secreting CD19 + B-cell clones) (Fig. 1E).

On further assessment of antibody cross-reactivity, the DENV vaccination induced a dominant DENV-2-specific clonal IgG response to envelope and virus-like particles (Supplementary Fig. 2A) with only limited or absent YF-17D or other orthoflaviviral cross-reactive IgG clones. The DENV-2–dominant profile is consistent with the established immunogenicity pattern of TAK-003, in which a majority of vaccine recipients develop a DENV-2–skewed response due to the vaccine’s DENV-2 backbone^16,20^. After YF-17D-204, the IgG cross-reactivity between heterologous envelope antigens increased (Fig. 1F). This broad clonal orthoflavivirus and clonal YF-17D-specific IgG pattern resembled control responses (Supplementary Fig. 2B).

### Potent YF virus-specific T-cells

**Fig. 2:**
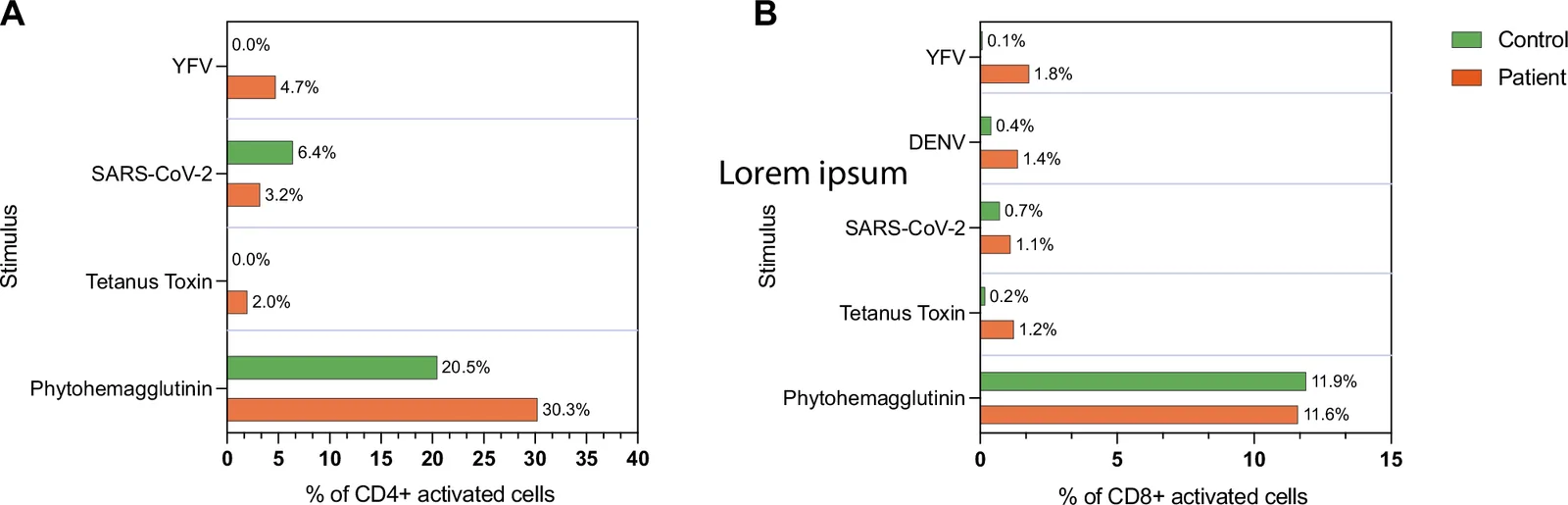
Orthoflavivirus-specific and control antigen T-cell responses. **A** Control patient (green) and patient (orange) AIM-positive (CD25/CD134/CD69/CD137) events within the CD4+ T cell compartment. **B** Control patient (green) and patient (orange) AIM-positive (CD69/CD137) events within the CD8+ T cell compartment.

We assessed the frequency of YF virus-specific T-cells 28 days after YF-17D-204 (D78, Fig. 2). Peripheral blood mononuclear cells were stimulated with a pan-YF select peptide pool (JPT, Berlin), containing 150 synthetic peptides covering HLA class I/II-restricted T-cell epitopes in capsid, envelope, non-structural proteins 1-5, and control antigens. After stimulation, activation-induced marker expression (CD25, CD69, CD134, and CD137 for CD4 + ; CD69 and CD137 for CD8+; gating strategy in Supplementary Fig. 3 and Supplementary Fig. 4) confirmed induction of YF virus-specific CD4+ and CD8+ T-cells (Fig. 2) with interferon-γ detected in the supernatant. Combined with normal responses to control conditions, this reflected normal T-cell functionality. A second TAK-003 vaccine was administered at D90 to complete the DENV vaccination as recommended.

## Discussion

This case highlights the status of the field regarding YF vaccination in thymectomized individuals and provides a clinical proof-of-concept approach to enable YF vaccination following thymectomy through a monitored proxy immunization strategy. After validating adaptive immune function to unrelated pathogens, safety and immune competence were assessed by administering the live-attenuated tetravalent DENV vaccine TAK-003 as an orthoflaviviral proxy, before YF-17D-204 vaccination. This stepwise evaluation of adaptive immune function confirmed intact humoral and cellular responses, normal control of live-attenuated orthoflaviviral replication, and no adverse events. These findings guided a shared decision to proceed with YF vaccination, which elicited broad, functional B- and T-cell immunity comparable to healthy vaccinees. Broad B-cell reactivity and cross-reactive antibodies after sequential DENV and YF vaccination indicate preserved germinal center function, class switching, and B-cell diversity. These are hallmarks of competent T-cell-dependent humoral immune maturation. Comparable clonal breadth to a control provides mechanistic evidence that thymectomy did not impair adaptive immunity diversification. Proxy vaccination with the DENV vaccine thus acts as an orthoflaviviral biological stress test, mirroring the adaptive pathways needed for YF-17D-204 control. This case illustrates how a live-attenuated orthoflavivirus proxy can individualize vaccine eligibility in patients for whom YF vaccination is otherwise contraindicated.

The selection of TAK-003 as an experimental live-attenuated orthoflaviviral proxy required a precise risk assessment against alternative live-attenuated orthoflavivirus options, particularly regarding the danger of ADE in a DENV-naive individual. Other live-attenuated candidates were ruled out due to safety and regulatory barriers; the JEV vaccine IMOJEV utilizes a YF 17D genetic backbone that carries the same risk of YEL-AVD post-thymectomy. Additionally, both IMOJEV and the standalone live JEV strain (SA14-14-2) are unlicensed in the Netherlands, and administering a live-attenuated version of a natively neurotropic pathogen would introduce an unquantifiable clinical risk in this patient. Crucially, the first-generation live dengue vaccine (CYD-TDV, Dengvaxia®) is heavily restricted because its structural-only design can prime naive individuals for severe disease via ADE upon natural exposure. In contrast, TAK-003 retains a complete non-structural viral architecture (NS1, NS3, NS5), which elicits a broad, polyfunctional CD8+ T-cell safety buffer against ADE^20^. Indeed, the IgM and IgG NS1 response to TAK-003 was empirically confirmed in our patient by longitudinal PMA serology data. Phase III studies confirm that vaccination in naive individuals remains highly effective and is heavily reassured by 4.5-year TIDES trial data showing no signal of increased severe disease or hospitalized illness upon natural heterologous exposure in thousands of baseline DENV-naive cohorts^15,16^. This robust safety profile underpins the current Dutch consensus for TAK-003 vaccination in dengue-seronegative travelers, advising vaccination for prolonged or repeated visits (≥3 months within a year) to high-risk endemic areas. Taken together, utilizing TAK-003 as a proxy vaccination served as the safest live biological stress test for this patient.

The role of the thymus in YEL-AVD remains uncertain and has limited evidence from clinical studies. Crucial early in life for establishing a diverse T-cell repertoire, the thymus undergoes age-related involution that reduces naive T-cell output and may underlie higher YEL-AVD risk in elderly^21^. Thymectomized adults show diminished thymic lymphocyte output and increased all-cause mortality, though infection risk is not elevated^22^. These data suggest that thymectomy alone does not cause generalized immunodeficiency, but warrants individualized evaluation for YF vaccination.

In the setting of over 540 million YF vaccinations since 1937^10^, our literature search (Supplementary Table 2) yielded only six individual YEL-AVD cases in relation to confirmed thymus disease, all in individuals with thymectomy for thymoma. An overview of these six cases is shown in Table 3A. Barwick et al.^6^ presented four cases of YEL-AVD in association with thymectomy for thymoma, of which two had been previously published^2,3^. DeSilva et al. published a fifth case of fatal YEL-AVD in a patient who appeared to have thymoma at autopsy and positive acetylcholine receptor antibodies one day before her death, consistent with myasthenia gravis^7^. Gnanadurai et al. performed a national review of reported YF vaccine incidents in the UK, in which they report one case of fatal YEL-AVD in a patient with prior thymectomy for thymoma^8^. Further details on this patient are missing. In addition, three other cases were identified for whom the occurrence of an associated thymus disorder was uncertain (Table 3B)^23–25^. Bae et al. described a non-fatal outcome of a YF-vaccine-associated neurotropic disease (YEL-AND) in a patient who was later found to have a thymoma, although the timing of this diagnosis after YEL-AND occurrence was not described. Isenman et al. described a patient who had a suspected thymoma on chest X-ray at hospital admission with YEL-AVD and subsequently died. No post-mortem analysis was done to confirm this. Le Hir et al. described a case of a fatal YEL-AVD in a patient who had unilateral ptosis, possibly indicating a feature of myasthenia gravis, a condition known associated with thymoma.

**Table 3:** A Clinical description of cases with confirmed thymus disorders before yellow fever (YF) vaccination and clinical outcome; B Clinical description of cases with possible thymus disorder before YF vaccination, or thymus disorder diagnosed after YF vaccination, with clinical outcomes

Report	Case	Country	Condition	Outcome
Martin (2001) Barwick (2004)	67-year-old female	USA	Thymectomy for a malignant thymoma about 2 years before vaccination	YEL-AVD, fatal
Barwick (2004)	70-year-old male	USA	Myasthenia gravis, and thymectomy for thymoma 20 years before vaccination	YEL-AVD, survived
Troillet (2001) Barwick (2004)	50-year-old male	Switzerland	Thymectomy for thymoma 8 years before vaccination	YEL-AVD, survived
Barwick (2004)	44-year-old male	Colombia	Thymectomy for thymoma 2 years before vaccination	YEL-AVD, fatal
DeSilva (2014)	60-year-old female	USA	Thymoma at autopsy, elevated acetylcholine receptor binding antibody in blood collected 1 day before death, indicative of myasthenia gravis	YEL-AVD, fatal
Gnanadurai (2022)	Unknown	UK	Thymectomy for thymoma (unknown how long before vaccination)	YEL-AVD, fatal
Bae (2008)	37-year-old female	Spain	Thymoma during follow-up (timing relative to vaccination unknown)	YEL-AND, survived
Isenman (2012)	66-year-old man	New Zealand	Suspected thymoma on X-ray at time of admission	YEL-AVD, fatal
Le Hir (2024)	54-year-old male	France	Intermittent unilateral ptosis (temporal relation unknown)	YEL-AVD, fatal

*YEL-AVD* yellow fever vaccine-associated viscerotropic disease, *YEL-AND* yellow fever vaccine-associated neurotropic disease.

Collectively, this pattern suggests that thymoma-associated immune dysregulation, not thymectomy itself, confers risk of YEL-AVD^2,3,6–8^. Myasthenia gravis, often linked to thymoma^6,7^, involves acetylcholine receptor antibodies affecting thymocyte survival^26^. Thymoma is also associated with other autoimmune diseases and Good’s syndrome, which features B-cell dysfunction^27,28^. Defective interferon signaling has likewise been implicated in YEL-AVD risk^14^ but was absent in our patient. Differentiating structural thymic removal from functional immune dysregulation is therefore critical when considering YF vaccination.

Prevention of YF virus infection remains the cornerstone in the absence of any approved antiviral drug. Off-label use of sofosbuvir, an NS5B nucleotide inhibitor used in hepatitis C virus infection, has in vitro activity against YF virus^29,30^ and potential effectiveness in outbreak settings^29^. A trial to repurpose sofosbuvir for YF virus infection treatment (SOFFA) is planned^31^. Interestingly, in the setting of a thymectomy, a recent report described a 30-year-old man with prior thymectomy for a benign thymic cyst who inadvertently received a YF vaccination and started sofosbuvir post-exposure prophylaxis 6 days afterwards without developing YEL-AVD while achieving seroconversion^32^.

With only alternative live-attenuated YF vaccines in phase 2/3 trial development (NCT05421611, NCT05011123) and no YF virus-specific treatment currently in late-stage trial development, optimizing existing YF vaccination strategies with live-attenuated vaccines can impact public health. Our approach suggests that integrated clinical and immunological markers can identify thymectomized individuals who safely tolerate a live-attenuated YF vaccination. The orthoflaviviral proxy-vaccination model provides a method for such assessment. Vaccination was justified by myasthenia gravis remission without immunosuppression, absence of thymoma, preserved B- and T-cell function, normal response to DENV vaccine with viral control, and absent interferon auto-antibodies. Proposed criteria (Supplementary Table 3) may guide such decisions, though broader validation is required. Outstanding questions include the relevance of potential orthoflavivirus cross-reactivity reducing YF viremia, which limits conclusions on DENV priming necessity^5^, and the long-term clinical significance of the pan-flaviviral IgG surge induced by YF vaccination after prior DENV vaccination. While these cross-reactive antibodies can theoretically influence the risk of ADE upon future natural heterologous exposure, they may also confer baseline cross-protection in vivo. The unproven long-term protection, the possibility that innate immune defects contribute to YEL-AVD risk, and restricted proxy immunization use in DENV-endemic regions remain further areas requiring study.

In conclusion, a monitored proxy orthoflavivirus vaccination offers an individualized immunologically guided pathway to expand YF vaccine eligibility after thymectomy, balancing the rare risk of viscerotropic disease against the increasing global need for YF protection amid rising outbreaks and global travel.

## Methods

### DENV serology

DENV–specific IgG and IgM were measured by using an enzyme-linked immunosorbent assay (Euroimmun); DENV NS1 antigen was measured by ELISA (Bio-Rad). YFV-specific IgM and IgG were measured by immunofluorescence (Flavi Mosaic, Euroimmun). All assays were performed following the manufacturer’s protocol.

### YF serology

The YF-PRNT is an in-house Plaque Reduction Neutralization Test specific for YF, testing for 80% YF neutralization. The range of the test is between a titer of 8–32, with a cutoff of ≥10 being considered protective against YF.

The titer is determined by serially diluting the patient-sera, incubating these dilutions with an in-house grown YF-17D stock for 1 hour and then suspended to Vero ATCC cells. A methylcellulose-DMEM overlay is added, and after 5 days of incubation, the plates are fixed and stained with YF-specific antibodies (Monoclonal Mouse IgG2a Yellow Fever Antibody (3576), Santa Cruz Biotechnology, Inc.). Read-out is done by using an automated analyzer (ImmunSpot UV Image Analyzer Series 6 Ultimate), which counts the plaques with the built-in software. By comparing plaque counts from the serum dilutions with plaque counts from a negative control (100% point), a neutralizing antibody titer can be calculated.

### Protein microarray slides preparation

Using a sciFlexArrayer SX (Scienion), each pad of a 64-pad UniSart Microarray slide (#16799, Sartorius) was coated with nanodrops of equal concentrations of dengue virus-like particles (VLP) of DENV1 Puerto Rico/US/BID-V853/1998, DENV2 Thailand/16681/84, DENV2 Sri Lanka D3/H/IMTSSA-SRI/2000/1266, and DENV4 Dominica/814669/1981 and orthoflavivirus envelopes of the aforementioned DENV2 and DENV4 strains, YF 17D-204-USA, JEV SA-14, WNV NY99 and ZIKV Suriname Z1106033, all from the Native Antigen Company, or TBEV Neudoerfl from Acro Biosystems. Each pad was also coated with NS1 proteins of DENV1 Nauru/Western Pacific/1974, DENV2 Thailand/16681/84, DENV3 Sri Lanka D3/H/IMTSSA-SRI/2000/1266, DENV4 Dominica/814669/1981, YF 17D-204, JEV SA-14, WNV NY99, and TBEV Neudoerfl, all from the Native Antigen Company. Additionally, pads were coated with SARS-CoV-2 D614G Spike (#40589-V08H8, Sino Biological) and Tetanus Toxoid (#BA108VS, Serion Immunologics) as control antigens.

### Orthoflaviviral-specific IgM/IgG detection on protein microarrays

Serum samples obtained at D7, D14, D28 and D50 after TAK-003 vaccination and D7, D14 and D28 after 17D-204 vaccination were 4-fold serially diluted in Blocker™ BLOTTO (Thermo Fisher), starting at a dilution of 1:10. Protein microarray (PMA) slides were blocked with Blocker™ BLOTTO (Thermo Fisher) for 1 hour at 37°C, after which diluted serum samples were added to the slides, and incubated again for 1 hour at 37°C. PMA slides were washed with PBS + 0.05% Tween-20, and incubated with a detection antibody Alexa Fluor 647 AffiniPure® Goat Anti-Human IgG or Cy™3 AffiniPure® Goat Anti-Human IgM (Jackson Immunoresearch) for 1 h at 37 °C. PMA slides were washed with PBS + 0.05% Tween-20 four times, followed by a single wash with distilled water, after which slides were air dried and scanned using an InnoScan 1100 scanner (Innopsys). Fluorescent signal on antigens was analyzed with Mapix software. Serum EC50 IgG or IgM titers were calculated using GraphPad Prism v10.

### B-cell culture and stimulation for IgG harvesting

CD19 + B-cells were isolated from PBMC at D7, D14, D28, and D50 after TAK-003 vaccination and D7, D14, and D28 after 17D-204 vaccination using magnetic EasySep™ Human CD19 Positive Selection Kit II (STEMCELL Technologies). B-cells were co-cultured at a density that was titrated to give clonal IgG reactivity on protein microarray (data not shown). Oligoclonal cultures of 200 to 1000 B-cells with 10 times the number of growth-arrested, 60 Gy irradiated CD40L-expressing fibroblasts (provided by J. Banchereau from The Jackson Laboratory for Genomic Medicine, Farmington, Connecticut, USA) in AIM-V AlbuMAX culture medium (Thermo Fisher) supplemented with 10% FBS, 100 U/mL penicillin, 0.1 mg/mL streptomycin (Capricorn Scientific), and 50 mM β-mercaptoethanol (Gibco) (B-cell medium), 50 U/ml interleukin 2 (IL-2; Novartis), 10 ng/ml IL-10 (Peprotech), 1 μg/ml Resquimod (InvivoGen) and 25 ng/ml IL-21 (Peprotech). After 3 days, culture supernatants were replaced with B-cell medium supplemented with 25 ng/mL IL-21 (PeproTech) for cell expansion and differentiation into antibody-secreting cells. 14 days after the initiation of cultures, culture supernatants were harvested and stored at −20 °C for analysis.

### B-cell IgG screening on protein microarrays

Antigen-specific IgG reactivity of B-cell culture supernatants was determined on protein microarray (PMA) slides. Slides were incubated with Blocker™ BLOTTO (Thermo Fisher) for 1 hour at 37°C and washed with PBS + 0.05% Tween-20. A mix of 1:1 of Blotto and B-cell culture supernatant was subsequently added to PMA slides and incubated for 1 hour at 37°C. PMA slides were then washed with PBS + 0.05% Tween-20 followed by fluorescent staining using a secondary detection antibody (Alexa Fluor 647 AffiniPure® Goat Anti-Human IgG, Jackson Immunoresearch) diluted 1:500 in Blocker™ BLOTTO. Stained slides were washed, dried, and scanned similarly to serum PMA slides. Mean fluorescence intensity (MFI) values per antigen were determined using Mapix software and averaged per duplo. Based on background reactivity in non-reactive culture supernatants, a threshold of 1500 MFI was set for positive IgG reactivity for all antigens.

### YFV-specific screening of B-cell-derived IgG

To test for B-cell culture supernatant reactivity to the YF-17D vaccine strain virus, Vero cells (ATCC) were cultured on 96-well flat-bottom culture plates (Greiner) with DMEM (Lonza) supplemented with penicillin/streptomycin, 10% Fetal Bovine Serum (FCS), sodium bicarbonate, and HEPES (10% FCS-DMEM). At 90% cell confluency, Vero cells were infected with the YF-17D strain at an MOI of 0.5 in 2% FCS-DMEM. After 72 h, the infection medium was removed, and the cells were washed with PBS. Cells were fixed with 4% PFA (Santa Cruz Biotechnology) for 30 minutes, washed with PBS, permeabilized with PBS supplemented with 0.1% Triton X-100 (Sigma Aldrich) for 10 minutes, and washed with PBS and blocked with PBS supplemented with 1% Bovine Serum Albumin (Blocking Buffer; BB) for 30 minutes at 37°C. Subsequently, a mix of 1:1 B cell culture supernatant and BB was added to the cells and incubated for 1 h at 37°C. Cells were washed with PBS + 0.05% Tween-20, and incubated with an Alexa Fluor 488 goat-anti-human IgG (Thermo Fisher) diluted 1:200 in BB for 1 h at 37°C. Subsequently, plates were washed with PBS and scanned for fluorescence at an Amersham Typhoon (Cytiva). Mean Fluorescence Intensity per well was calculated with ImageJ software. An MFI of >14,000 was considered positive for reactivity to YF virus based on background fluorescence on wells without B cell culture supernatants. Each positive well was confirmed using confocal microscopy and Zen 2010 software (Zeiss) (data not shown).

### Measurement of antigen-specific T-lymphocytes

Antigen-specific T-lymphocytes were measured using a commercially available kit (Act-T4 Cell™, Cytognos, Spain) that is based on the so-called ‘OX40 assay’. Briefly, heparinized whole blood was diluted 1:1 with RPMI-1640 (Gibco, Paisley, UK) culture medium supplemented with 10% fetal calf serum (Gibco), 2 mM L-glutamine (BioWhittaker), and penicillin and streptomycin (100 IU/mL; BioWhittaker), or PBMCs were suspended in the above-mentioned medium. Diluted whole blood or PBMC were aliquoted into 500 μL volumes in sterile capped 5 mL polystyrene flow cytometry tubes. The following conditions were used for each assay: no exogenous stimulation (negative control/DMSO control), PHA (2.5 μg/mL), Staphylococcal enterotoxin B (SEB; Sigma-Aldrich) (0.5 μg/mL), Tetanus toxoid (1 μg/mL), *Candida albicans* lysate (4 μg/mL), CMV lysate (2 μg/mL), VZV lysate (1 μg/mL), Dengue PepPool (Mabtech) (2–4 μg/mL), and Pan-Yellow Fever Select peptide pool (JPT) (0.5–2 μg/mL). After 24 or 44 h stimulation in a humidified atmosphere of 5% CO_2_, samples were vortexed and 100 μL of each condition were stained with the 4-antibody mixture provided in the Act-T4 Cell™ kit (CD3-PerCP-Cy5.5, CD4-FITC, CD25-APC, and CD134-PE, Cytognos), CD8-APC-H7(BD Biosciences), CD45-PO (Invitrogen), CD69-BV421 (Biolegend) and CD137-PE-Cy7 (Biolegend) and incubated for 15 minutes at room temperature. During staining, the assay was protected from light. After incubation, 1 mL of erythrocyte lyse-non-wash solution provided in the Act-T4 Cell™ kit was added to each tube, mixed, and incubated for 10 minutes at room temperature, still protected from light. After lysis of erythrocytes, 10,000 CD3-positive events were acquired on a flow cytometer (FACSLyric) and analyzed with BD FACSuite software (BD Biosciences), and T-cell subpopulations were counted.

### Literature search strategy and selection criteria

We identified data for the systematic literature review through a comprehensive search of the PubMed/Medline, Embase, Web of Science, and Cochrane databases from database inception to 1 May 2025. The literature review was specifically performed to identify cases of YF vaccination associated with thymus disorders.

The exact search strategies utilized for each database are provided in Supplementary Fig. 5. Only articles published in English were included. Additional searches were conducted in Google Scholar to identify relevant literature from alternative sources, and a bibliographic search of all included studies was performed to capture further eligible reports. Citations were imported into Covidence systematic review software (Veritas Health Innovation, Melbourne, Australia) for subsequent screening, review, and data extraction.

## Supplementary information

Supplementary Information

## Data Availability

The datasets generated and/or analyzed during the current study are not publicly available due to institutional data protection policies, but are available from the corresponding author on reasonable request.
