# Supplementary material for: Proxy immunization enabling yellow fever vaccination after thymectomy
Source: NPJ Vaccines. 2026 Jun 29;11:193. doi: 10.1038/s41541-026-01520-x (PMC13683385; doi:10.1038/s41541-026-01520-x)
Supplement: Supplementary file 1 [file 41541_2026_1520_MOESM1_ESM.pdf]

SUPPLEMENTARY INFORMATION TO

# Proxy Immunization Enabling Yellow Fever Vaccination after Thymectomy

**Emilie C. Rijnink*[,1,2], Muriel Aguilar-Bretones*[,3], Rory D. de Vries[3], Sharon Veenbergen[4], Daniël G. Aynekulu Mersha[3], Corine H. Geurts van Kessel[3], Marion P.G. Koopmans[3], Pieter A. van Doorn[5], Lennert Slobbe[1,2], Rik A. Brooimans[4], Gijsbert P. van Nierop[3] & Casper Rokx[1,2]**

[1]Department of Internal Medicine, Section Infectious Diseases, Erasmus University Medical Center, Rotterdam, Netherlands. [2]Department of Medical Microbiology and Infectious Diseases, Erasmus University Medical Center, Rotterdam, Netherlands. [3]Department of Viroscience, Erasmus University Medical Center, Rotterdam, Netherlands. [4]Laboratory Medical Immunology, Department of Immunology, Erasmus University Medical Center, Rotterdam, Netherlands. [5]Department of Neurology, Erasmus University Medical Center, Rotterdam, Netherlands.
*both authors contributed equally

## Contents

**SUPPLEMENTARY TABLES** ........................................................................ 2

**SUPPLEMENTARY FIGURES** .................................................................... 5

**SUPPLEMENTARY TABLES**

**Supplementary Table 1. Baseline T-cell activation after *ex vivo* stimulation of whole blood**

| | Unstimulated | PHA | SEB | CA | TT | PPD | DT | CMV | HSV1 | HSV2 | VZV | SARS-CoV-2 |
|---|---|---|---|---|---|---|---|---|---|---|---|---|
| %$CD134^+/CD25^+$ $CD4^+$ T cells | 0.3 | 27.7 | 11.4 | 7.1 | 3.8 | 4.9 | 0.6 | 0.4 | 0.3 | 0.2 | 4.9 | 8.0 |

*Abbreviations: CA, Candida albicans; CMV, cytomegalovirus; DT, diphtheria toxin; HSV, herpes simplex virus; PHA, phytohemagglutinin; PPD, purified protein derivative; SARS-CoV-2, severe acute respiratory syndrome coronavirus 2; SEB, staphylococcal enterotoxin B; TT, tetanus toxoid; VZV, varicella-zoster virus.*

**Supplementary Table 2. Results of literature search**

| Database searched | Platform | Years of coverage | Records | Records after duplicates removed |
|---|---|---|---|---|
| Medline ALL | Ovid | 1946 - 2025 | 21 | 21 |
| Embase | Embase.com | 1971 - 2025 | 47 | 25 |
| Web of Science Core Collection* | Web of Knowledge | 1975 - 2025 | 24 | 6 |
| Cochrane Central Register of Controlled Trials** | Wiley | 1992 - 2025 | 1 | 1 |
| Additional Search Engines: Google Scholar*** | | | 50 | 44 |
| **Total** | | | **143** | **97** |
| | | | | |
| **Screening Phase** | **Process** | | | |
| Title & Abstract Screening | Screened for initial relevance | | 97 | |
| | Excluded as irrelevant | | 54 | |
| Full-Text Review & Eligibility | Reports assessed for eligibility | | 43 | |
| | Excluded after full-text review† | | 37 | |
| Alternative Sourcing | Added via bibliographic search | | 2 | |
| Final Inclusion | **Studies selected for data extraction‡** | | **8** | |

**Science Citation Index Expanded (1975-present) ; Social Sciences Citation Index (1975-present) ; Arts & Humanities Citation Index (1975-present) ; Conference Proceedings Citation Index- Science (1990-present) ; Conference Proceedings Citation Index- Social Science & Humanities (1990-present) ; Emerging Sources Citation Index (2005-present); **Manually deleted abstracts from trial registries: ***Google Scholar was searched via "Publish or Perish" to download the results in EndNote. No other database limits were used than those specified in the search strategies. †Full-text exclusion breakdown: Calculated to balance screening attrition. Out of the 43 full-text articles assessed, 37 were excluded following deep review, leaving 6 database-derived papers that directly progressed to final extraction. ‡Final inclusion details: The definitive case results for these 8 finalized studies are provided in Tables 3A and 3B of the main text, which include 2 studies identified through a manual bibliographic search during the full-text review stage.*

**Supplementary Table 3: Proposed criteria for YF vaccine administration post-thymectomy**

| **Clinical criteria** |
|---|
| 1. Age <65 years |
| 2. No abnormal infectious burden<br>*Defined as: Absence of recurrent, severe, or atypical infections that are otherwise unexplained or exceed expected rates in the general population* |
| 3. Absence of auto-immune disease |
| 4. In case of myasthenia gravis, adequate clinical remission without the need of immunosuppressive medication* |
| 5. No/limited reactogenicity to childhood vaccination and proxy vaccination** |
| **Pathological criteria** |
| 1. Absence of thymoma preferred<br>2. With a resected thymoma, caution is warranted and any clinical signs of a thymoma associated immune disorder must be absent |
| **Laboratory criteria** |
| 1. Normal immunoglobulin (IgG, IgA, IgM) levels |
| 2. Childhood vaccine response present<br>*Defined as: assessment of antibody levels to e.g. tetanus toxoid, diphtheria toxoid, Haemophilus influenzae type b (Hib), measles, mumps, or rubella.* |
| 3. Appropriate serologic response and limited viremia to proxy vaccination** |
| 4. Auto-antibodies to type 1 IFN preferably excluded |
| 5. Normal CD4+/CD8+T-cell and B-cell counts and T-cell function |
| 6. Normal cellular immune proliferation response to proxy vaccination* |

**Detectable isolated anti-acetylcholine receptor antibodies after thymectomy require a thorough clinical work and prolonged monitoring to confirm clinical remission. **Proxy vaccination: vaccination with a live-attenuated vaccine where we suggest a flaviviral vaccine such as TAK-003 in a patient with a dengue naive immune system. Consider serological screening for antibodies first.*

## SUPPLEMENTARY FIGURES

**Supplementary Figure 1. Non-structural protein 1 antibody responses.**

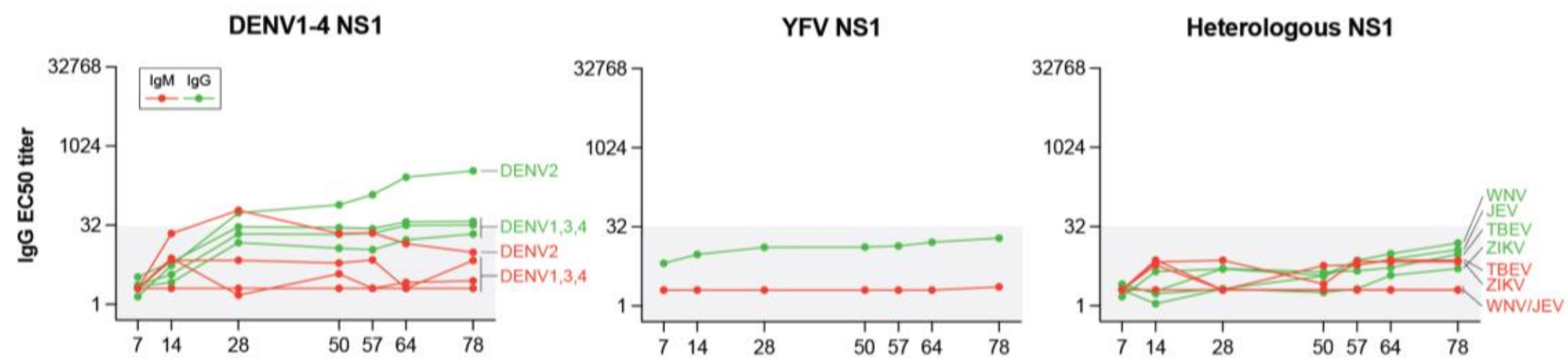


*The 50% effective concentration (EC50) serum titers for IgG (green) and IgM (red) towards non-structural protein 1 (NS1) of dengue virus serotype 1-4 (DENV1-4; left panel), yellow fever virus (YFV; middle panel), and the heterologous orthoflaviviruses West-Nile virus (WNV), Japanese encephalitis virus (JEV), tick-borne encephalitis virus (TBEV) and zika virus (ZIKV) were determined (right panel) using protein microarray analysis.*

**Supplementary Figure 2. Clonal orthoflavivirus-specific B-cell analysis**

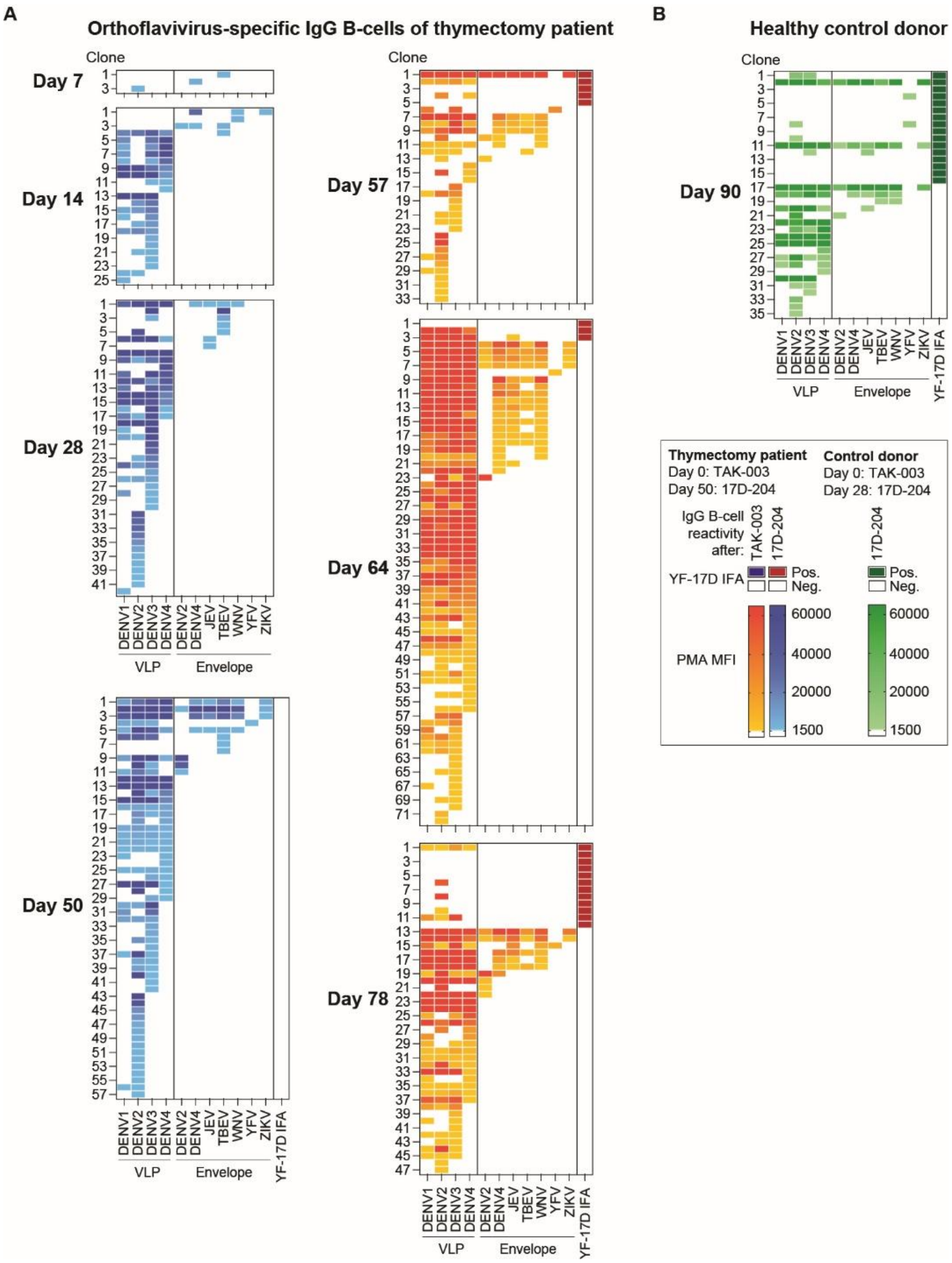


*A) Peripheral blood-derived CD19+ B-cells isolated at day 7, 14, 28, 50, 57, 64 and 78 of the thymectomy patient were cultured at limiting density to ensure clonal reactivity towards orthoflavivirus antigens. Culture supernatants were tested for IgG reactivity towards dengue virus serotype 1-4 (DENV1-4) virus-like particles (VLP), and envelope (E) of DENV2 and 4, Japanese and tick-borne encephalitis virus (JEV, TBEV), West-Nile virus (WNV), yellow fever and Zika virus (WNV, ZIKV) on protein microarray (PMA) and yellow fever vaccine virus (YF-17D) infected cells using immunofluorescent assay (IFA). The mean fluorescent intensity (MFI) of reactive cultures is indicated in blue shades after TAK-003 vaccination (day 0) and orange shades after YF-17D-204 vaccination (day 50). IgG reactive cultures for YF-17D IFA are shown in dark blue or red in the right column. B) Clonal IgG B-cell reactivity of a control donor that received TAK-003 on day 0 and YF-17D on day 28, and was sampled on day 90. The MFI of PMA is shown in green shades and positive YF-17D IFA in dark green.*

**Supplementary Figure 3. Gating strategy for CD4+ and CD8+ T-cells**

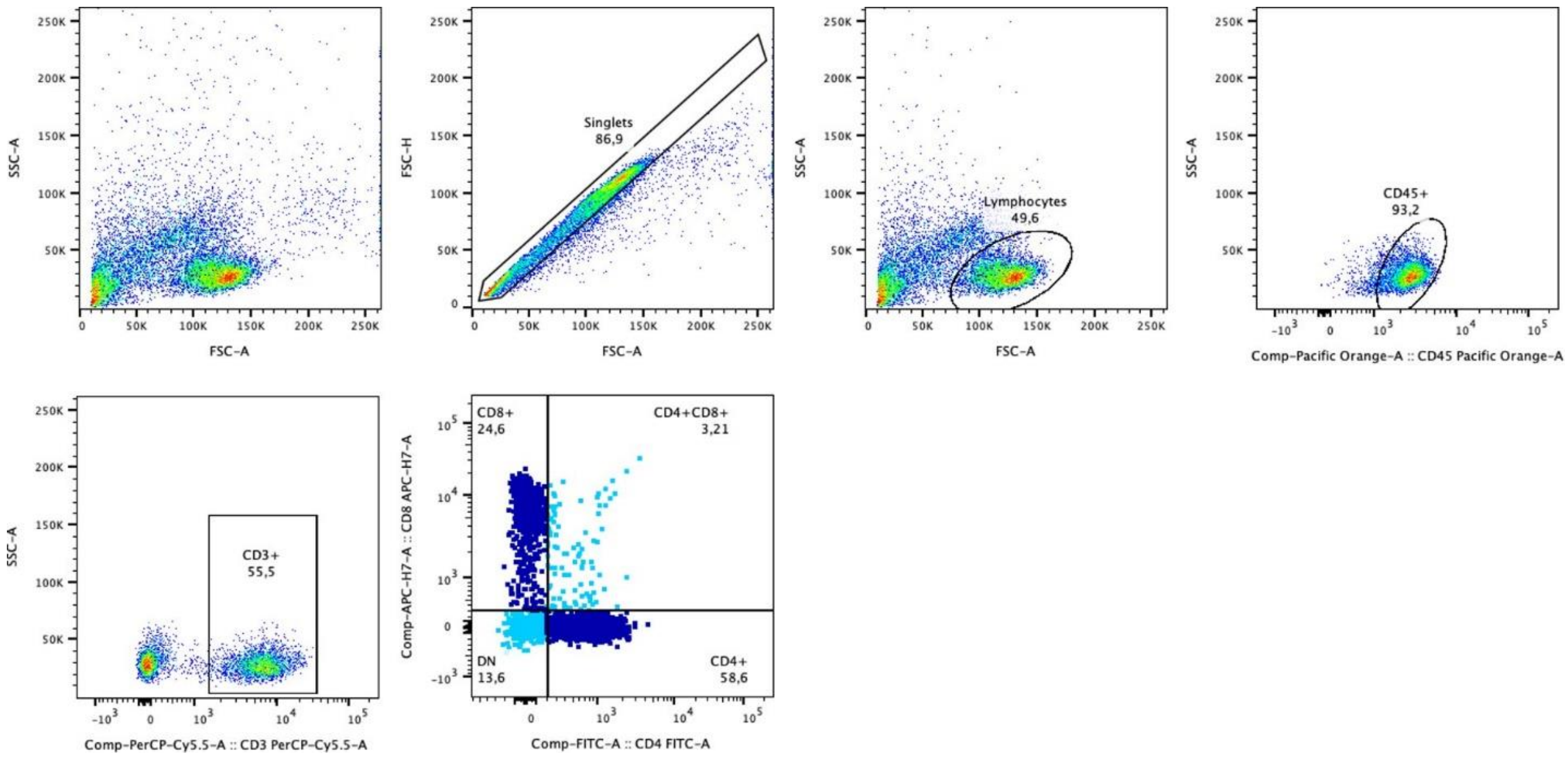


*Whole peripheral blood mononuclear cells were gated for single cell events. Lymphocytes were gated based on size and granularity and further selected for analysis based on CD45 expression. Within the CD45+ lymphocytic population, those that expressed CD3+ (T cells) were further classified into CD8+ and CD4+ expression. CD4+ and CD8+ T-cell events were used for further flow cytometry analysis.*

**Supplementary Figure 4. Gating strategy for orthoflavivirus-specific T-cells**

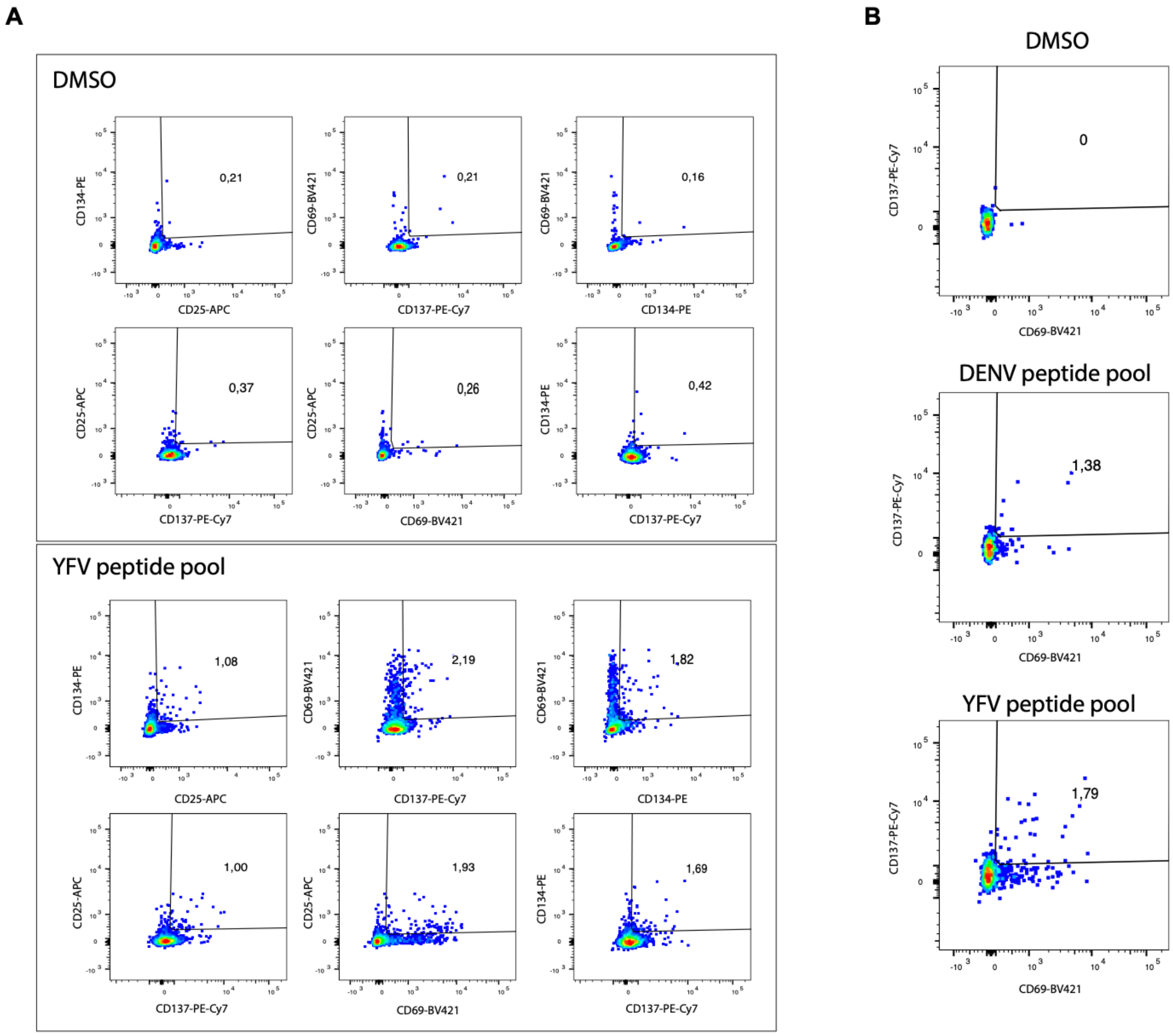


*A) CD4+ T cells gated following the gating strategy described in Figure S2 were further characterized for AIM markers (CD25, CD134, CD69 and CD137) expression. Double positive events for every combination of AIM markers were merged into a single percentage of activation with an OR gate, also known as Boolean gating. B) CD8+ T-cells gated following the gating strategy described in Figure S2 were further analyzed for CD69 and CD137 double positivity.*

**Supplementary Figure 5. Search strategy per database**

**Medline**
(exp Thymus Gland/ OR exp Thymectomy/ OR exp Thymus Neoplasms/ OR (thymus* OR thymic* OR thymon* OR thymom* OR thymectom*).ab,ti,kf.) **AND** (exp *"Vaccines, Live, Unattenuated"/ OR exp Yellow Fever Vaccine/ OR (((yellow*) ADJ3 (fever*) ADJ6 (vacci*)) OR yfvax OR yf-vax).ab,ti,kf. OR (((live*) ADJ4 (vacci*))).ti.) **NOT** (exp animals/ NOT humans/)

**Embase**
('thymus'/exp OR 'thymus surgery'/exp OR 'thymic neoplasm'/exp OR (thymus* OR thymic* OR thymon* OR thymom* OR thymectom*):ab,ti,kw) **AND** ('live vaccine'/exp/mj OR 'yellow fever vaccine'/exp OR (((yellow*) NEAR/3 (fever*) NEAR/6 (vacci*)) OR yfvax OR yf-vax):ab,ti,kw OR (((live*) NEAR/4 (vacci*))):ti) NOT ((animal/exp OR animal*:de OR nonhuman/de) NOT ('human'/exp))

**Web of Science**
TS=(thymus* OR thymic* OR thymon* OR thymom* OR thymectom*) **AND** (TS=(((yellow*) NEAR/2 (fever*) NEAR/5 (vacci*)) OR yfvax OR yf-vax) OR TI=(((live*) NEAR/3 (vacci*)))) **NOT** TS=((animal* OR rat OR rats OR mouse OR mice OR murine OR dog OR dogs OR canine OR cat OR cats OR feline OR rabbit OR cow OR cows OR bovine OR rodent* OR sheep OR ovine OR pig OR swine OR porcine OR veterinar* OR chick* OR zebrafish* OR baboon* OR nonhuman* OR primate* OR cattle* OR goose OR geese OR duck OR macaque* OR avian* OR bird* OR fish*) NOT (human* OR patient* OR women OR woman OR men OR man))

**Cochrane**
((thymus* OR thymic* OR thymon* OR thymom* OR thymectom*):ab,ti,kw) **AND** ((((yellow*) NEAR/3 (fever*) NEAR/6 (vacci*)) OR yfvax OR yf NEXT/1 vax):ab,ti,kw OR (((live*) NEAR/4 (vacci*))):ti)

**Google Scholar**
thymus|thymic|thymoma|thymectomy 'yellow fever vaccin|vaccins|vaccination'|'YF vaccine|vaccines|vaccination' 'adverse event|events|effect|effects'|complication|complications
